# Clear cell variant of oral squamous cell carcinoma: case report and review

**DOI:** 10.4322/acr.2021.388

**Published:** 2022-07-18

**Authors:** Rutuja Narsing Mukkanwar, Sangeeta Palaskar, Rasika Pawar, Darshana Rajesh Shah

**Affiliations:** 1 Sinhgad Dental College and Hospital, Department of Oral and Maxillofacial Pathology, Pune, Maharashtra, India

**Keywords:** Carcinoma, Squamous Cell, Mouth Neoplasms, Sarcoma, Clear Cell, Squamous Cell Carcinoma of Head and Neck

## Abstract

The clear cell variant of Oral Squamous Cell Carcinoma (OSCC) is an uncommon histological variant. Kuo first discovered it in the skin, and Frazier et al. found it in the oral cavity. We know of only nine cases of clear cell variant of OSCC reported in the literature till now. The present case is in a 60-year-old male patient with an ulcer on the left posterolateral border of the tongue. The patient had a history of chewing tobacco for 22 years. Clinical examination showed features of malignant ulcer associated with pain and discomfort. Histopathological examination revealed sheets and islands of atypical epithelial cells with clear cytoplasm, nuclear and cellular pleomorphism, and few keratin pearls in the connective tissue suggesting OSCC. Various special stains were performed to identify clear cells. Periodic Acid Schiff-Diastase (PAS-D) and Mucicarmine stains showed positive and negative reactions in clear cells, respectively. Immunohistochemical (IHC) analysis for cytokeratin (AE1/AE3) showed diffuse positivity in clear cells and other epithelial cells. Based on special stains and IHC markers, we confirmed the diagnosis as a clear cell variant of OSCC. This variant is rare and presents diagnostic challenges. It is said to be aggressive in nature. More such cases should be reported to understand its biological behavior and prognosis.

## INTRODUCTION

Oral Squamous Cell Carcinoma (OSCC) is the eighth most common cancer worldwide.^1^ It is the most common type of malignancy affecting the head and neck region,^2^ accounting for 96% of all oral malignancies.^1^ Etiologic agents for OSCC are primarily tobacco (smoking and smokeless) and alcohol consumption. Other risk factors include poor oral hygiene, malnutrition, immunosuppression, high exposure to sunlight, syphilis, sanguinaria, trauma, candida infection, human papillomavirus (HPV) infection, and chronic irritation.^3^ OSCC is most common in people between 45 and 75 years,^1^ with 377713 cases occurring every year.^4^

Histopathologically, OSCC is graded as well-differentiated, moderately differentiated, or poorly differentiated squamous cell carcinoma.^5^ Conventional OSCC may show several histopathological variants, aggregating about 10%–15% of all squamous cell carcinomas (SCC).^6^ These variants include Basaloid Squamous Cell Carcinoma, Verrucous Carcinoma, Spindle Cell Carcinoma, Adenoid Squamous Cell Carcinoma, Adenosquamous Carcinoma, and Lymphoepithelioma.^7^

Clear Cell variant of OSCC (CCOSCC) is a very rare histological variant.^5^ Kuo first described it in the skin in 1980,^8^ and there are only nine reported cases in the oral cavity to date. Although the WHO has not recognized the clear cell variant in classifying oral cavity tumors, the literature discusses this entity’s occurrence, nature, and biological behavior.^9^ Different authors have suggested that “clear cell changes” could be a progressive process seen in advanced cases of OSCC, signifying its aggressive nature with possible early metastasis leading to poor prognosis.^5^ ^,^ ^10^

This case report describes the case of a rare variant of OSCC as CCOSCC of the tongue with clinical and histopathology findings and literature review.

## CASE REPORT

**Figure 1:**
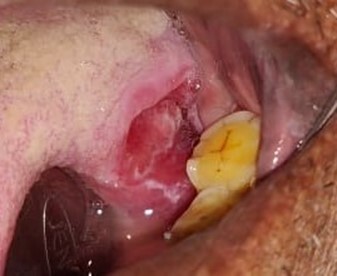
Gross view of the ulcer on the posterolateral border of the tongue.

A 60-year-old male patient reported an ulcer on the posterolateral border of his tongue for the past 6 months. There was pain associated with the ulcer while eating and speaking. The patient had a habit of chewing tobacco for 22 years. A clinical examination revealed a sharp lingual cusp on 36 and 37. On inspection of the lesion, a single crater-like ulcer was present on the left posterolateral border of the tongue, measuring approximately 1.5 cm × 2.0 cm in size, oval in shape, with a velvety red base (Figure 1).

On palpation, the lesion’s borders were indurated with mild tenderness. Lymph nodes were not palpable, so we did not demonstrate MRI or PET scan for any distant metastasis. Given the lesion site, the patient did not undergo radiography before having a shaving type of incisional biopsy. We submitted the specimen for histopathological examination.

**Figure 2:**
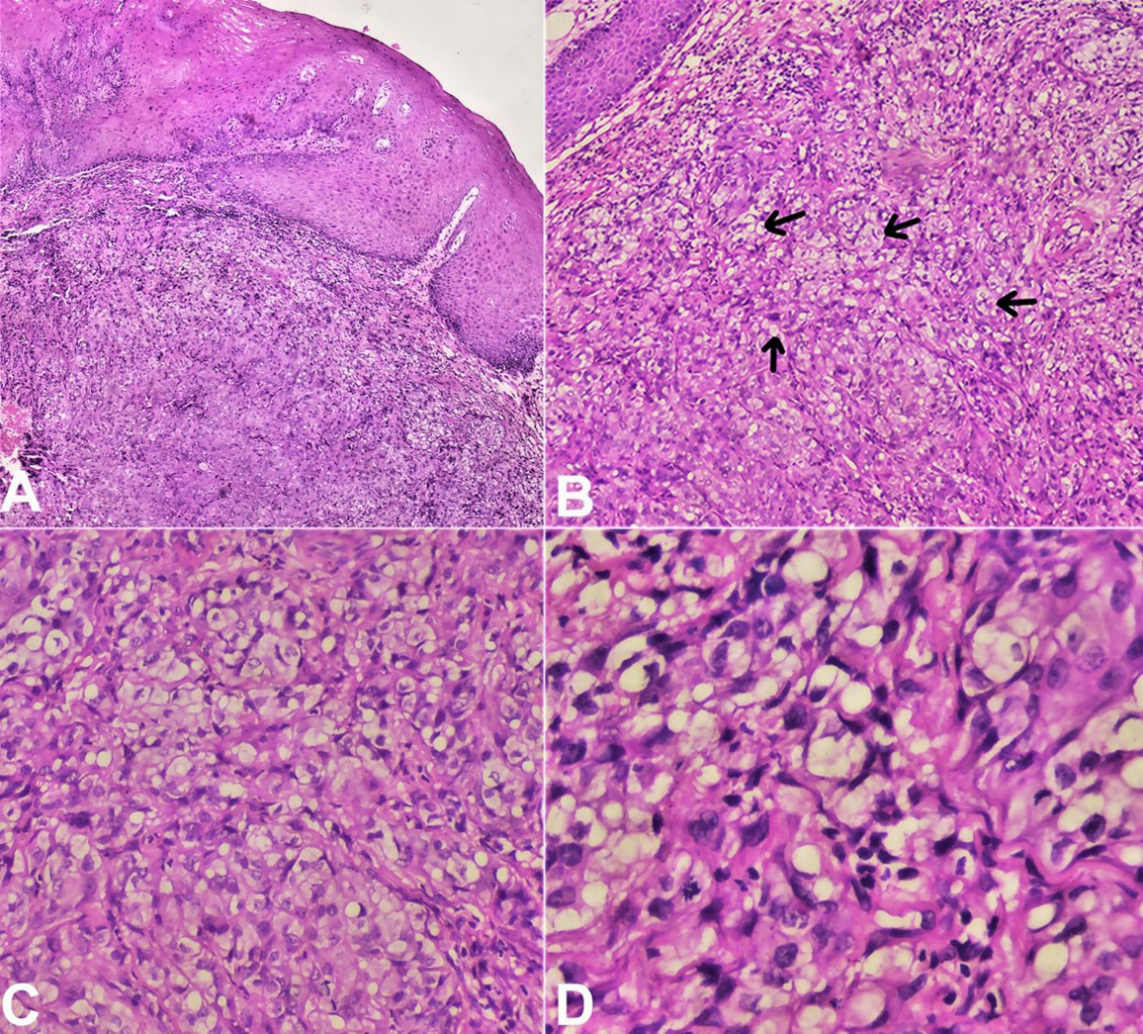
Photomicrographs of the biopsy. **A –** dysplastic surface epithelial cells with large, pleomorphic, and hyperchromatic nuclei infiltrating the connective tissue stroma (H&E,4X); **B –** connective tissue stroma infiltrated with tumor cells (Arrow) (H&E, 10X); **C –** connective tissue stroma containing dysplastic clear cells along with epithelial cells (20X); **D –** clear cells with cellular and nuclear pleomorphism (40X).

Gross examination showed a single soft tissue bit measuring approximately 0.7 cm × 0.5 cm × 0.3 cm in size, creamy color, with a soft consistency. The whole tissue was taken for processing and stained with Hematoxylin and Eosin (H&E). Microscopic examination showed ulcerated stratified squamous surface epithelium and cellular connective tissue stroma. The epithelium exhibited hyperplasia, hyperkeratinization, cellular, and nuclear atypia with a breach in the basement membrane and invading the underlying connective tissue. Connective tissue stroma contains atypical epithelial cells in sheets with few keratin pearls in focal areas. Islands of round to polygonal-shaped cells with clear cytoplasm occupied almost 50% of the stromal tissue (Figure 2).

**Figure 3:**
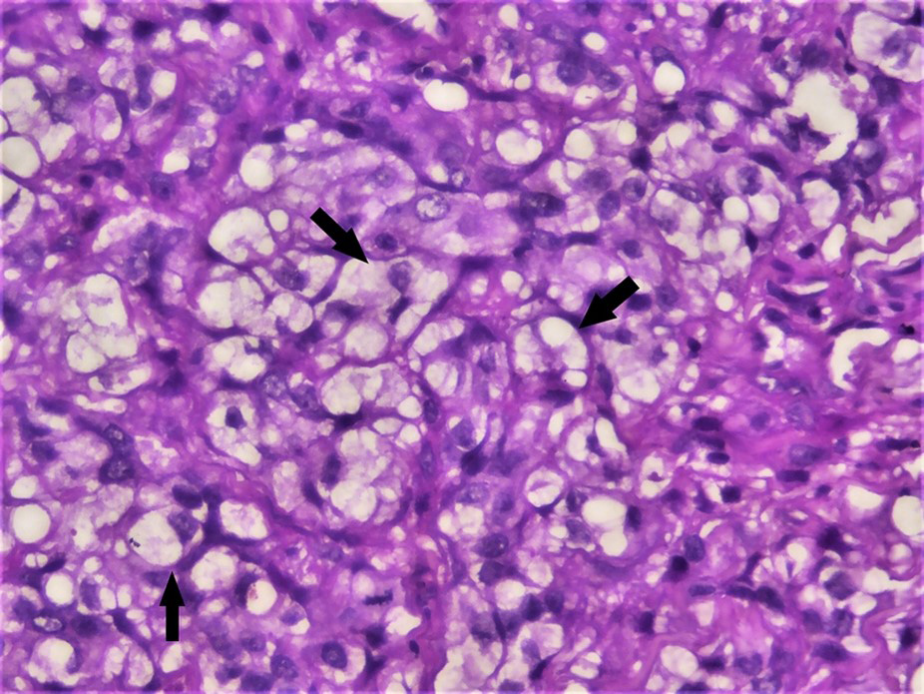
Photomicrograph of the biopsy. Positive PAS diastase staining in clear cells (arrow) (40X).

The diagnosis of well-differentiated squamous cell carcinoma was rendered based on the above findings. Additional investigations were performed to understand the nature and significance of histopathologically evident clear cells. We performed special stains such as Periodic Acid Schiff (PAS) diastase and mucicarmine to rule out clear cells of various origins. The tumor cells rich in glycogen showed strong positivity for PAS diastase (Figure 3).

**Figure 4:**
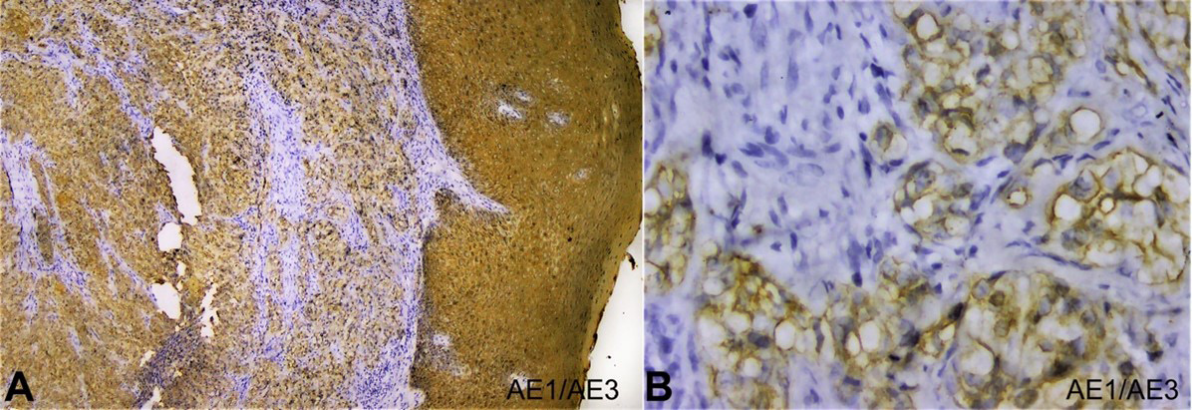
Photomicrograph of the biopsy -**A** and **B –** Immunohistochemical staining for cytokeratin (AE1/AE3) showing strong immunoreactivity in the epithelium and epithelial cells present in the connective tissue; **A –** (4X) and **B –** (40X).

Whereas the mucicarmine stain was negative, suggesting the absence of mucin, thus, excluding salivary gland neoplasms. The diagnosis of CCOSCC was then further confirmed by immunohistochemical staining with cytokeratin (AE1/AE3) marker. AE1/AE3 was strongly positive in the epithelium and the dysplastic epithelial island, keratin pearls, and clear cells in connective tissue stroma suggesting malignancy of epithelial origin (Figure 4).

The diagnosis was given as a Clear Cell Variant of Squamous Cell Carcinoma. The patient was referred to the cancer hospital for treatment. We advised complete excision of the lesion along with partial glossectomy and radiotherapy. Unfortunately, we have lost the further follow-up of the case.

## DISCUSSION

A clear cell is one in which the cytoplasm appears empty when stained with H&E.^10^ They are broadly classified as Physiologic and Pathologic (Algorithm 1).^9^

**Algorithm 1:**
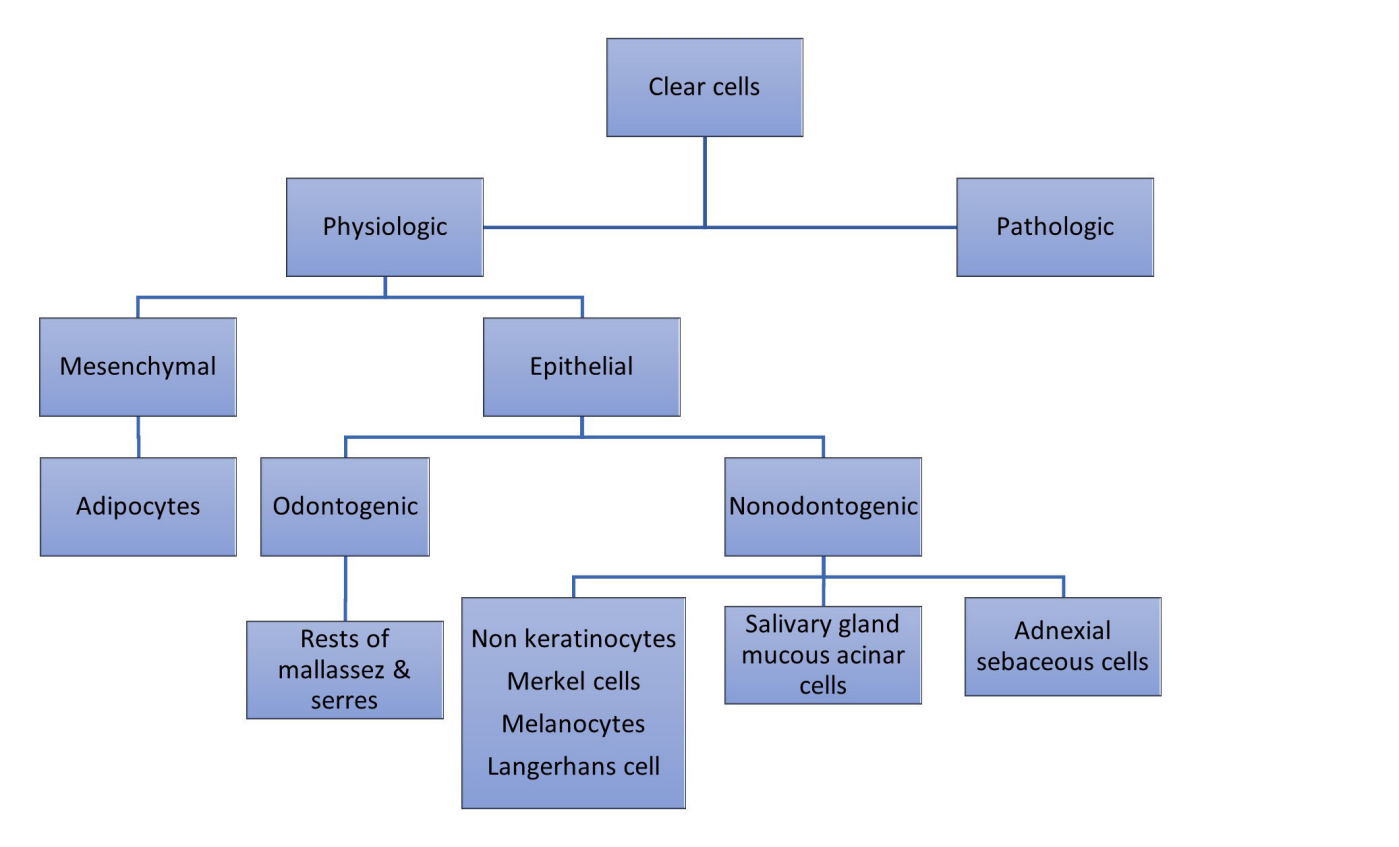
Algorithm 1. Classification of clear cells.

The cells’ clear appearance can be due to the content present in the cytoplasm, accumulated intracytoplasmic structures, or artifactual changes in the cytoplasm (Table 1).^9^ ^,^ ^11^

**Table 1:** Causes of clearing of cells

**Content of cytoplasm**	**Accumulation of intra-cytoplasmatic structures**	**Artifactual changes in the cytoplasm**
Mucopolysaccharides, immature zymogen granules	Water, glycogen, mucin	Fixation and histologic techniques
Intermediate filaments	Lipids	
Paucity of cytoplasmatic organelles	Phagocytized foreign material	
	Hydropic degeneration	

The clear cell lesions of the oral cavity can be categorized as salivary gland origin, odontogenic origin, and metastatic lesion. Salivary gland tumors with clear cell changes are clear cell myoepithelial carcinoma, acinic cell carcinoma, mucoepidermoid carcinoma, epithelial-myoepithelial carcinoma, and hyalinizing clear-cell carcinoma. Odontogenic clear cell lesions are clear odontogenic ghost cell tumors, clear cell odontogenic carcinoma. Metastatic lesions include carcinoma from renal cells, lung, liver, and breast. All these clear cell lesions can be considered differentials for CCOSCC.^10^ ^,^ ^12^

Special stains such as PAS with or without diastase rule out the various origins of clear cells such as adnexal, and salivary gland. Mucicarmine and alcian blue are used for mucin, and Sudan black for lipid. Along with special stains, various IHC markers such as cytokeratin type 8, 18, 19 for epithelial origin, Smooth Muscle Actin (SMA) and S100 for myoepithelial origin, S100 for melanocytic tumor, Epithelial membrane antigen (EMA), Carcinoembryonic Antigen (CEA) and P63 for adnexal origin are required to confirm the origin of clear cells in the clear cell lesions.^10^

Corbalán-Vélez et al.^13^ suggested that clear cells are common in SCCs after reviewing 249 cases of cutaneous SCC. They described two histologic distribution patterns of clear cells, one with clear cells and adnexal differentiation associated with Bowen’s disease and one with clear cells surrounding keratin pearls associated with actinic keratosis and positive for p16 suggesting HPV as an essential factor. Based on the percentage of clear cells from the tumor and adjacent area, histologic variables were obtained, i.e., cases without clear cells, clear cells less than 25%, between 26% and 50%, between 51% and 75%, and more than 75% and believed that clear cells are common in SCCs. However, they occur more in advanced SCC cases, suggesting a secondary phenomenon or clonal evolution.^14^

In the present case, we discussed a variant of OSCC composed of approximately 50% of the cells with clear cytoplasm, suggesting a clear cell variant.

There are only nine cases of CCOSCC in English literature (Table 2).^5^ ^,^ ^10^ ^-^ ^12^ ^,^ ^15^ ^-^ ^19^

**Table 2:** Table containing previously reported cases and present case of Clear Cell Variant of Squamous Cell Carcinoma

**Ref**	**Age/ Sex**	**Site and Presentation**	**Etiology**	**IHC Markers**	**Staining**	**Prognosis**
Kumar et al.^15^	70/ F	Lobulated growth 1: Maxillary anterior region in between central incisors Lobulated growth 2: Edentulous area of mandibular right first molar	Pan chewing with areca nut, lime, and tobacco	Positive – EMA, CK8, CK18	Negative – PAS, Mucicarmine	Died within 2 months
				Negative – Vimentin, S-100, HMB 45		
Frazier et al.^11^	59/ F	Exophytic lesion on the left mandibular gingiva extending from the buccal vestibule to floor of the mouth; Unhealed extraction socket	–	–	Positive – PAS	Lost to follow-up
					Negative – PAS diastase, Mucicarmine	
Nainani et al.^16^	52/ M	Irregular diffuse erythematous ulcero-proliferative growth on the left side of buccal mucosa.	No tobacco/ alcohol history	Positive – CK8, CK18	Negative – PAS, Mucicarmine, Oil red O	Died within 3 months
				Negative – Vimentin, S-100		
Romañach et al.^17^	60/ F	Ulcerated swelling in the posterior buccal mucosa extending to the soft palate.	–	Positive – CKAE1/AE3, p63	Positive – PAS	No recurrence after 12 months of surgery
				Negative – Vimentin, CD10		
Kaliamoorthy et al.^5^	35/ F	Non-healing ulcer involving left posterior lateral border of the tongue and lingual vestibule	No tobacco/ alcohol history	Positive – CK AE1/ AE3	Negative – PAS, Mucicarmine	–
				Negative – Vimentin, EMA, HMB 45.		
Devi et al.^18^	55/ M	an ulcerated swelling on Left posterior region of maxilla	–	Positive – CK, EMA	Negative – PAS, Mucicarmine	–
				Negative – S-100, Vimentin		
Khoury et al.^19^	66/ F	Ulcerative mass of the left lateral tongue extending anteriorly to the floor of the mouth	–	Positive – Pancytokeratin, CK5/6, and p63	Positive – PAS	3 months after surgery, metastasis to left lung
				Negative − S-100, Calponin, SMA		
Kakoti et al.^10^	59/ M	Exophytic growth in the right upper jaw	–	Positive – CK	Negative – PAS	–
				Negative – S-100, EMA		
Ramani et al.^12^	42/ F	a soft-tissue growth with erythematous and non-scrapable irregular white patches on the left alveolar mucosa in relation to 35, 36, and 37	–	Positive – CK	Negative – PAS, Mucicarmine	Lost to follow-up
				Negative – S-100, EMA, SMA, CD 117		
Index case	60/ M	Non-healing ulcer on posterolateral border of tongue	Tobacco chewing	Positive – CK	Positive – PAS diastase	Lost to follow-up
					Negative – Mucicarmine	

CK: cytokeratin; EMA: Epithelial membrane antigen; F: female; HMB: Human melanoma black; IHC: Immunohistochemical; M: male; PAS: Periodic acid Schiff; Ref: reference; SMA: Smooth muscle actin).

Six of the nine reported cases occurred in females; the remaining three were male patients, and this case is a male patient. Four out of nine cases affected the mandibular gingiva, three affected the maxilla, and only two affected the tongue’s lateral border. In this case, the lesion was on the posterolateral border of the tongue, making it the third case of CCOSCC affecting the lateral border of the tongue per the literature search. The clinical feature of previously reported cases showed the following observations: two presented with exophytic growth, five presented with ulceroproliferative growth, one with lobulated growth, and the remaining one case as a non-scrapable, irregular white patch. The current case was presented with an ulcer.

Of the nine cases, only one patient had a history of pan chewing with areca nut, lime, and tobacco. The patient had no deleterious habit in two cases, and the remaining cases reported no habit history. In the present case, the patient had a 22-year habit of chewing tobacco; he also had sharp cusps at 36 and 37.

Few authors mentioned the treatment and follow-up of the cases. Two patients died within 2–3 months after diagnosis; one of them reported metastasis to the lung, and the other reported local recurrence within 6 months after the first surgery without recurrence after 12 months of the second surgery. The prognosis of the present case is not known as we lost the patient’s follow-up. Per the review of previous cases, the clear cell variant is aggressive and requires extensive management. Long-term follow-up is advisable to prevent recurrences. There is a knowledge gap about the exact pathophysiology of CCOSCC, suggesting the need for a detailed study of this pathology. Molecular studies are required to understand the biological, clinical, and histopathological significance of these cases.

## CONCLUSION

CCOSCC is a rare entity and should be carefully diagnosed as it is said to be aggressive. Special staining and IHC are recommended for diagnostic confirmation. The prognosis is unclear due to a lack of literature. More research is required to understand the pathophysiology, biological behavior, treatment options, and prognosis. As it is not a rare variant in the skin and the oral variant could have been overlooked, it may be essential to add this to the WHO classification.
